# Supplementary material for: Behavioural plasticity and the transition to order in jackdaw flocks
Source: Nat Commun. 2019 Nov 15;10:5174. doi: 10.1038/s41467-019-13281-4 (PMC6858344; doi:10.1038/s41467-019-13281-4)
Supplement: Supplementary file 1 — Supplementary Information [file 41467_2019_13281_MOESM1_ESM.pdf]

Supplementary Information

Behavioural plasticity and the transition to order in jackdaw flocks

Ling et al.

Supplementary Figures

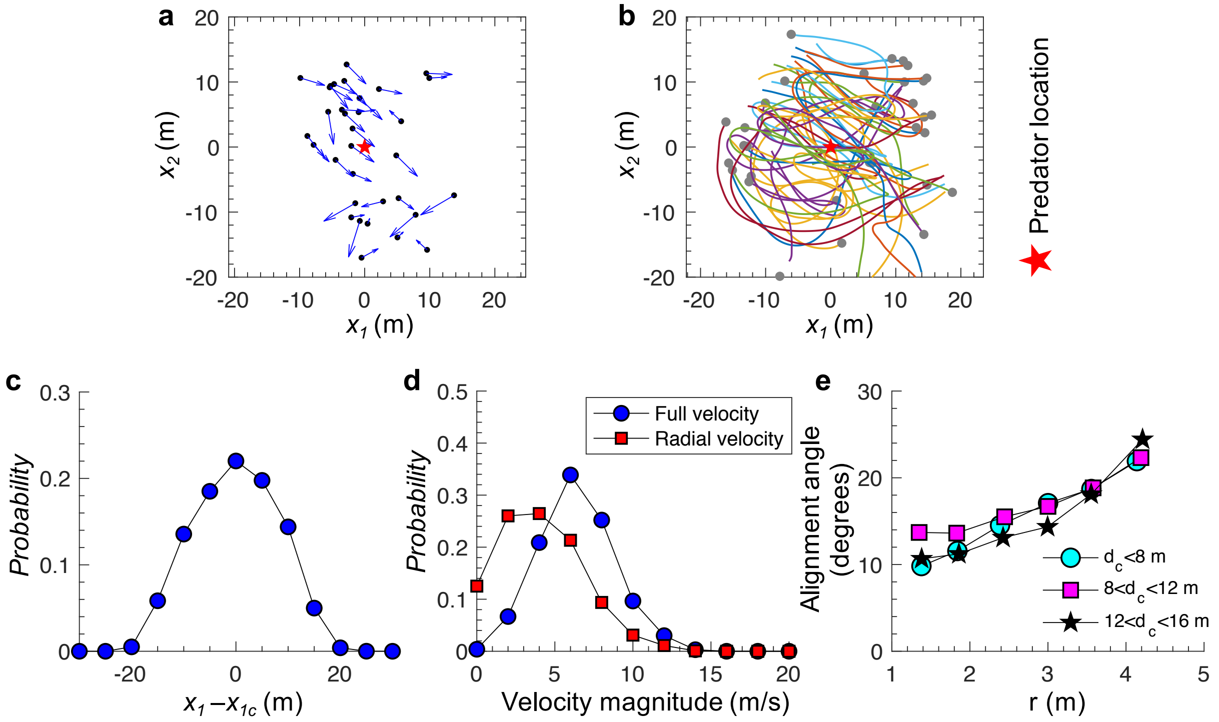

Supplementary Figure 1 | Alignment in mobbing flocks is not due to geometric effects. a. Full velocities and spatial distribution of individual birds at one selected time frame. **b.** The trajectories for the birds shown in **a**. In **a** and **b** the model predator is located at (0,0), and positions and velocities are projected onto a horizontal plane. **c.** Probability density function of the horizontal distance of a bird to the predator model x_1-x_{1c}, where x_{1c} is the location of model predator. **d.** Probability density functions of the full velocity and the velocity component in the direction of the model predator. **e.** Alignment angle as a function of r for three different ranges of d_c, where r is the distance between two birds and d_c is the distance of the focal bird to the predator. Figures **a**-**d** are for mobbing flock #M03, and the same patterns hold for all other cases. Figure **e** used data from all mobbing flocks.

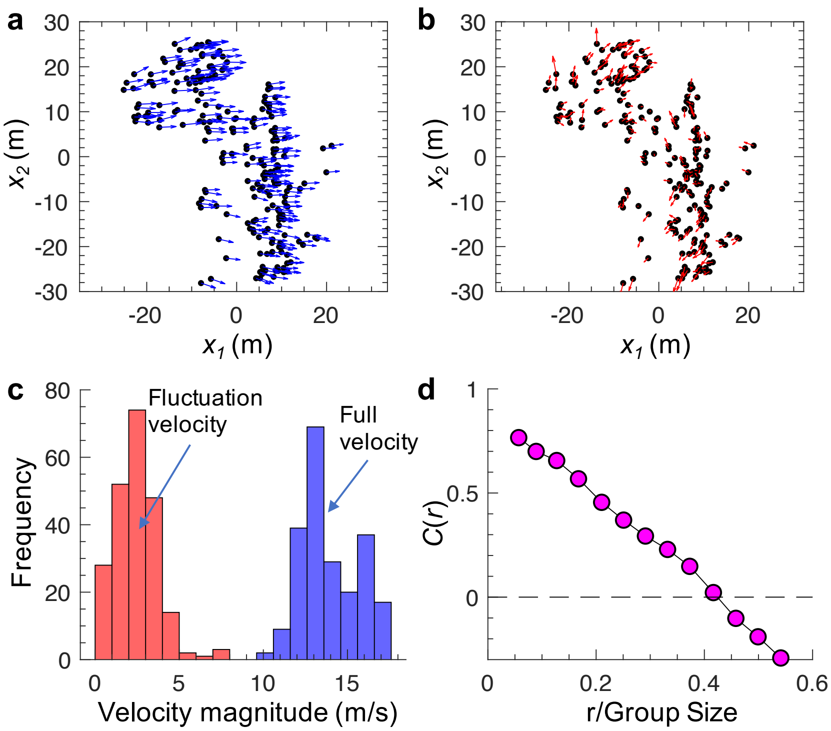

Supplementary Figure 2 | Velocity fluctuation in transit flocks. a. Full velocities and spatial distribution of individual birds in transit flock #01 at one selected time frame. **b.** Fluctuation velocities u' corresponding to **a**. **c.** Histograms of the magnitudes of full velocities and fluctuation velocities corresponding to **a** and **b**. **d.** Correlation function $C(r)$ of the velocity fluctuations shown in **b**. The group size is calculated as the averaged furthest distance between birds.

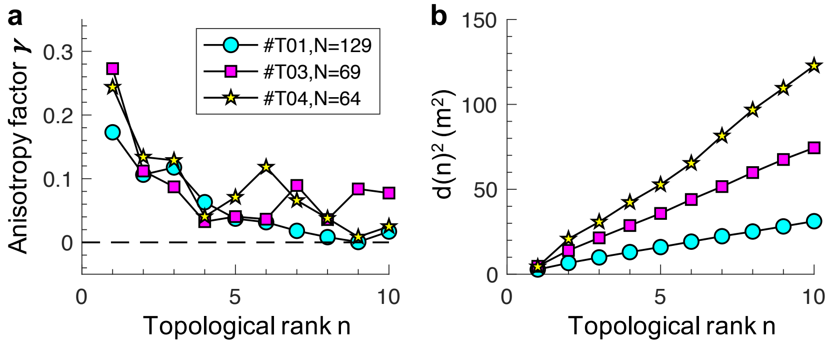

Supplementary Figure 3 | Topological interactions in transit flocks. a. The anisotropy factor γ as a function of topological rank n for transit flocks #01, 03, and 04. **b.** The average distance to the nth nearest neighbour, $d(n)$, as a function of n. N denotes the average number of birds in the flocks. Data for other transit flocks are not shown due to the small group sizes.

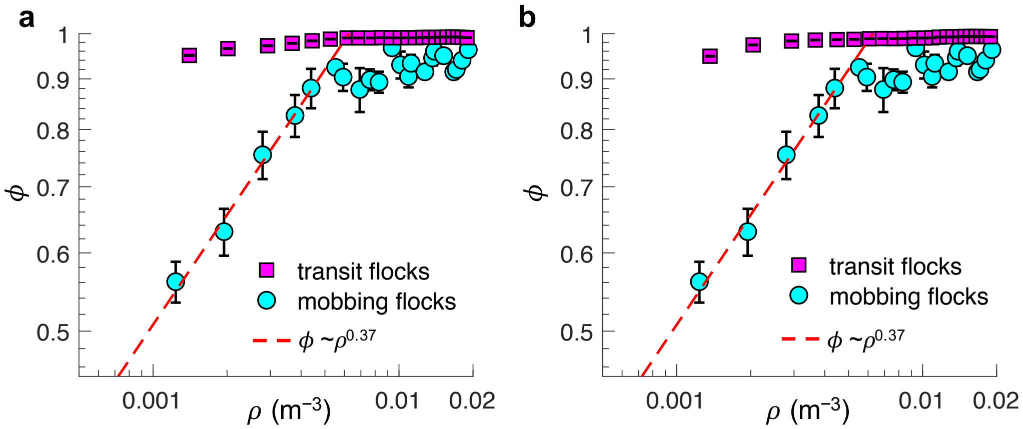

Supplementary Figure 4 | Relationship between group density and group order. For the mobbing flocks, the results are same as those presented in Fig. 3. For the transit flocks, results are calculated by choosing local subgroups of (**a**) one focal bird and its 9 nearest neighbours; and (**b**) one focal bird and its 19 nearest neighbours embedded in a larger flock.

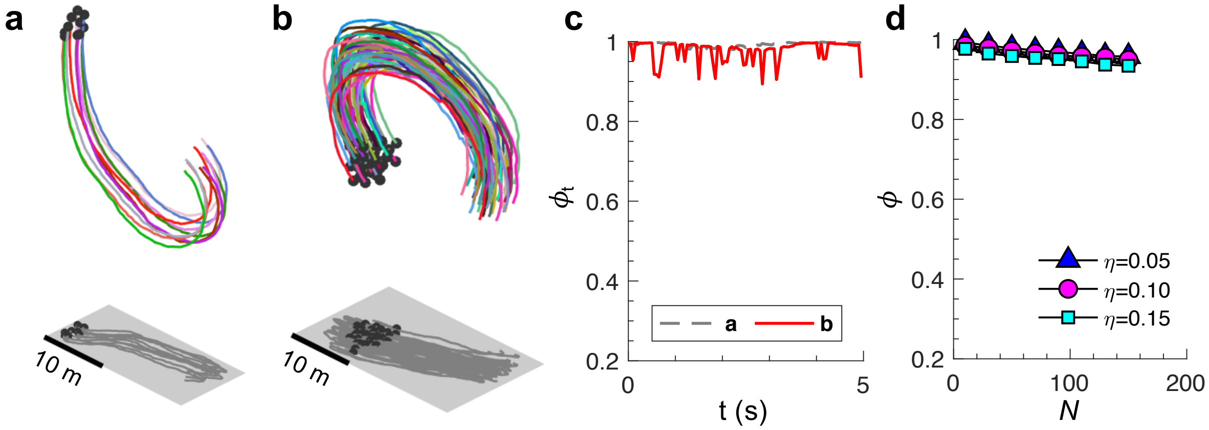

Supplementary Figure 5 | Model with topological interactions. a. A sample modelling result at small group size (N=10). **b.** A sample modelling result at large group size (N=50). **c.** Time-variation of ϕ_t for the cases shown in **a** and **b**. **d.** Group order ϕ as a function of group size N at three different noise levels.

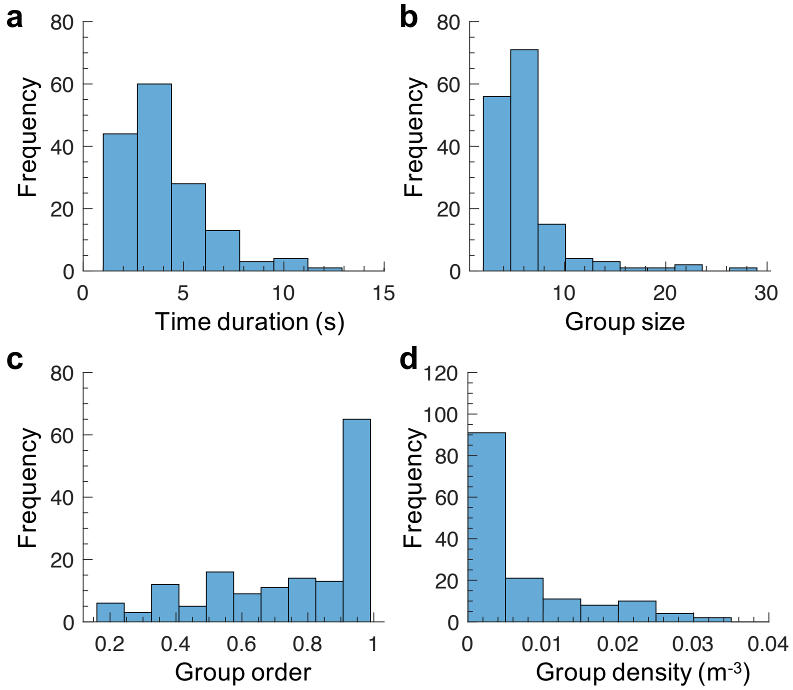

Supplementary Figure 6 | Statistics for 154 groups selected from the recorded mobbing flocks. a. Time duration. **b.** Group size. **c.** Group order. **d.** Group density.

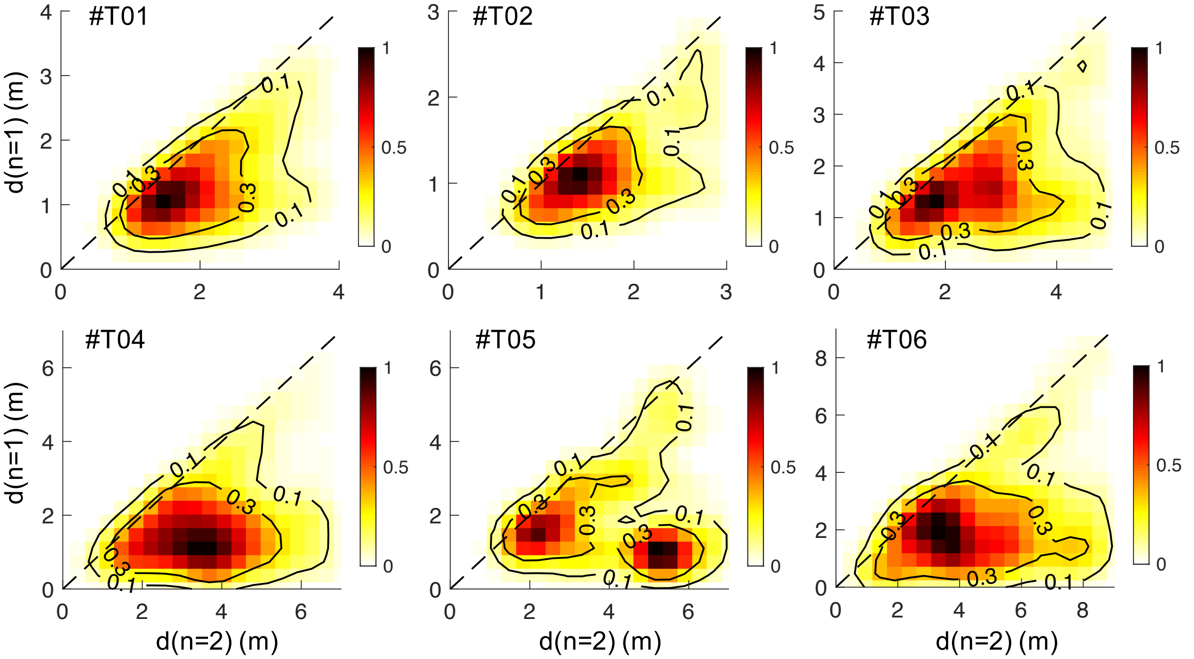

Supplementary Figure 7 | Evidence of pairwise social relationships in transit flocks. Joint probability density functions (PDFs) of $d(n=1)$, the distance to the nearest neighbour, and $d(n=2)$, the distance to the second-nearest neighbour, for transit flocks (#T01 to T06). All PDFs have a region of high probability where $d(n=1)$ remains nearly constant regardless of $d(n=1)$ indicating the existence of pairwise subgroups in transit flocks.

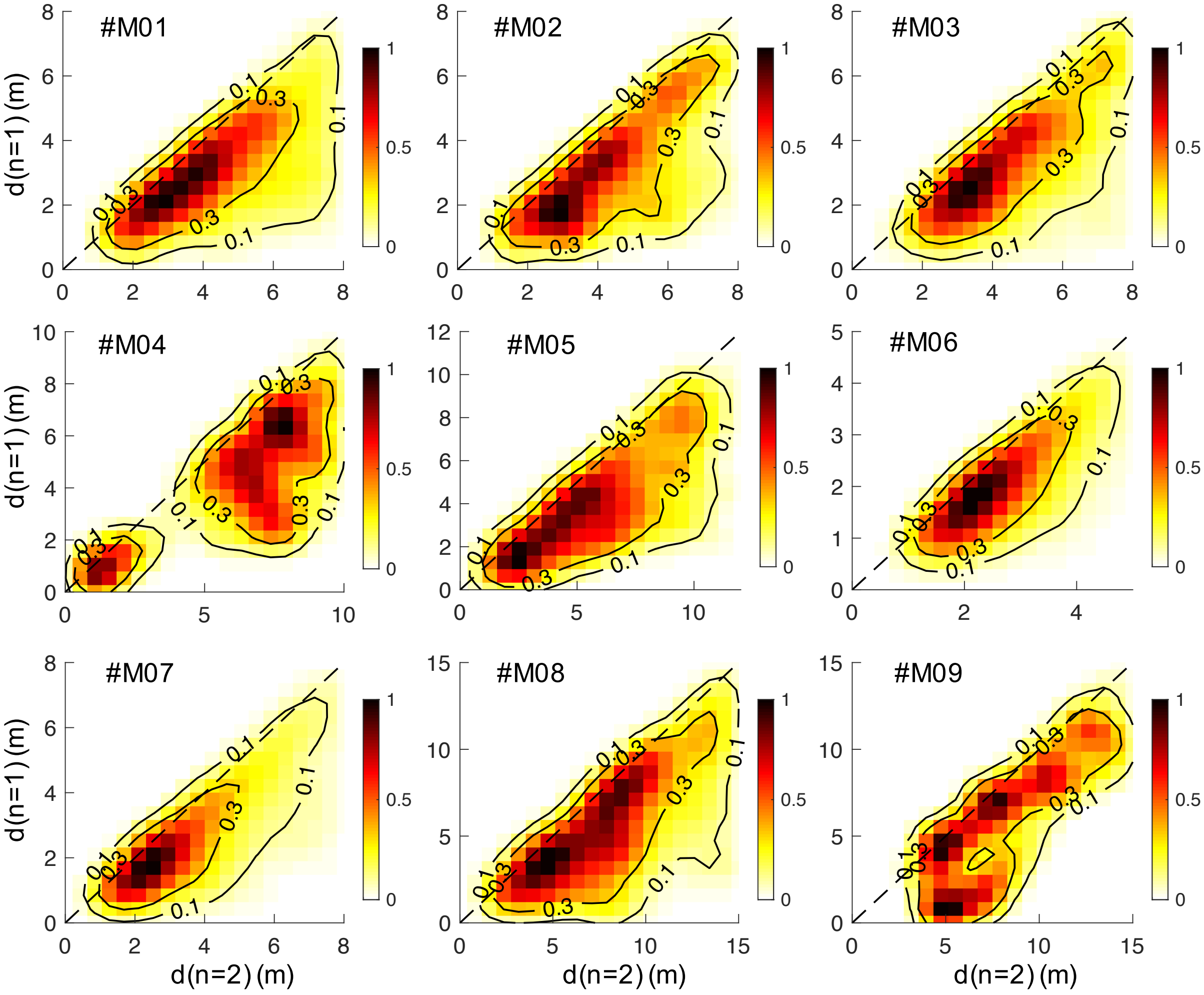

Supplementary Figure 8 | Lack of pairwise social relationships in mobbing flocks. Joint probability density functions (PDFs) of *d*(*n*=1), the distance to the nearest neighbour, and *d*(*n*=2), the distance to the second-nearest neighbour, for mobbing flocks (#M01 to M09). Data for mobbing flock #M10 is not shown due to the small group size. All PDFs show only one region of high probability where *d*(*n*=1) increases with *d*(*n*=2) indicating a lack of pairwise subgroups in mobbing flocks.

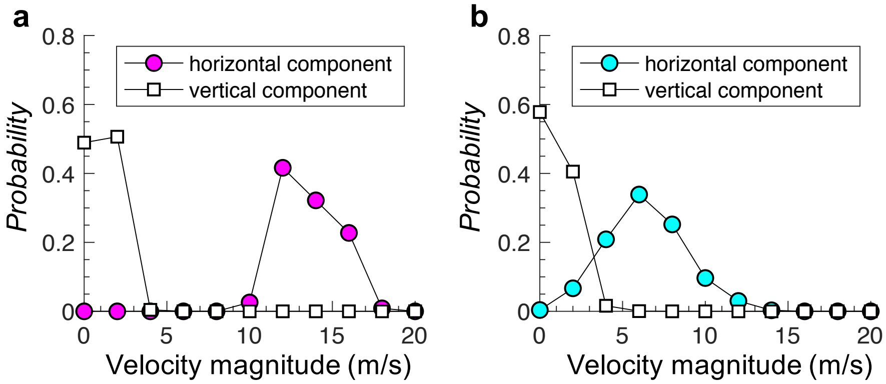

Supplementary Fig. 9. | Distributions of the velocity magnitude in the horizontal and vertical directions. a. Transit flocks. **b.** Mobbing flocks.

Supplementary Tables

Supplementary Table 1 | Statistics of 10 mobbing flocks and 6 transit flocks.

Event number	Recording during (s)	Group size	Group order	Nearest neighbour distance (m)	Flight speed (m/s)	Velocity in gravity direction (m/s)
Mobbing flocks recorded between May 2018 and July 2018						
#M01	124.0	10 ± 6	0.70 ± 0.25	6.1 ± 3.0	7.4 ± 1.7	0.1 ± 1.0
#M02	105.0	7 ± 6	0.71 ± 0.20	5.9 ± 2.0	6.9 ± 1.5	0 ± 1.0
#M03	130.7	10 ± 7	0.52 ± 0.22	6.2 ± 1.9	6.5 ± 2.3	0 ± 1.2
#M04	99.0	5 ± 1	0.70 ± 0.21	6.7 ± 2.2	7.1 ± 1.2	0.2 ± 1.1
#M05	89.0	4 ± 1	0.70 ± 0.23	5.9 ± 1.8	6.5 ± 1.9	0.5 ± 1.1
#M06	157.6	49 ± 43	0.71 ± 0.20	4.4 ± 2.3	6.9 ± 2.0	0.1 ± 1.1
#M07	114.3	14 ± 14	0.64 ± 0.21	6.6 ± 2.5	6.4 ± 2.7	0.2 ± 1.2
#M08	122.1	7 ± 3	0.57 ± 0.25	7.9 ± 2.8	7.9 ± 2.6	0 ± 1.3
#M09	88.4	4 ± 1	0.53 ± 0.25	9.9 ± 2.5	8.1 ± 1.2	0.1 ± 1.1
#M10	30.5	4 ± 1	0.80 ± 0.11	6.5 ± 3.6	7.4 ± 2.9	0.1 ± 1.5
Transit flocks recorded between Dec 2017 and March 2018						
#T01	7.5	124 ± 82	0.98 ± 0.01	2.1 ± 0.8	13.7 ± 1.7	–0.5 ± 1.2
#T02	3.3	31 ± 7	0.95 ± 0.03	1.9 ± 0.6	9.5 ± 3.0	0.4 ± 1.7
#T03	5.8	69 ± 27	0.99 ± 0.00	2.2 ± 0.3	12.6 ± 1.8	–0.8 ± 1.2
#T04	5.8	64 ± 17	0.98 ± 0.01	2.2 ± 0.3	12.6 ± 1.8	–0.8 ± 1.2
#T05	3.3	28 ± 3	0.99 ± 0.00	2.5 ± 0.3	11.2 ± 1.5	–0.4 ± 1.0
#T06	5.0	53 ± 9	0.92 ± 0.03	2.8 ± 0.3	10.0 ± 1.2	–0.8 ± 0.8

The values provided in the table are the means and standard deviations. The group size within a single flock varies due to birds entering and leaving the measurement volume during the data recording.

Supplementary Discussion

To test whether our finding for mobbing flocks could potentially result simply from birds flying in circles around the model predator, we built a two-dimensional circular flight model and compared its results with the empirical observations (we thank an anonymous reviewer for this suggestion). In this model, the agent's velocity is prescribed as $v_x \sim \sin(\theta + \text{noise})$ and $v_y \sim \cos(\theta + \text{noise})$. Although this simple model can capture the results shown in Fig. S1e (that is, that the alignment angle increases with metric distance between agents), it does not reproduce the results in Fig. S1d (that the radial velocity of birds with respect to the predator is on the same order of magnitude as the flight speed).
