# Supplementary material for: Behavioural plasticity and the transition to order in jackdaw flocks
Source: Nat Commun. 2019 Nov 15;10:5174. doi: 10.1038/s41467-019-13281-4 (PMC6858344; doi:10.1038/s41467-019-13281-4)
Supplement: Supplementary file 3 — Description of Additional Supplementary Files [file 41467_2019_13281_MOESM3_ESM.docx]

**Description of Additional Supplementary Files**

Supplementary Movie 1: “Original images captured by one of the four cameras and the reconstructed birds’ 3D movement trajectories for transit flocks #01 to 06.”

Supplementary Movie 2: “Original images captured by one of the four cameras and the reconstructed birds’ 3D movement trajectories for mobbing flocks #01 to 05.”

Supplementary Movie 3: “Original images captured by one of the four cameras and the reconstructed birds’ 3D movement trajectories for mobbing flocks #06 to 10.”
